# Data of real-world reference scores for EORTC QLQ-C30 and QLQ-BR23 at baseline in women with early breast cancer and other breast diseases

**DOI:** 10.1016/j.dib.2022.108347

**Published:** 2022-06-03

**Authors:** Pimrapat Gebert, Adam David Dordevic, Robert Roehle, Anna Maria Hage, Maria Margarete Karsten

**Affiliations:** a Berlin Institute of Health at Charité – Universitätsmedizin Berlin, Charitéplatz 1, Berlin 10117, Germany; b Institute of Biometry and Clinical Epidemiology, Charité – Universitätsmedizin Berlin, Berlin, Germany; c Department of Gynecology with Breast Center, Charité – Universitätsmedizin Berlin, Berlin, Germany

**Keywords:** Reference scores, Routine data, Breast cancer, DCIS, Fibroadenoma, EORTC, Health-related quality of life, eCRF, Web-based PRO, ePRO, DCIS, Ductal carcinoma in situ, eCRF, electronic clinical report form, ePRO, electronic Patient Reported Outcomes, EORTC, European Organisation for Research and Treatment of Cancer

## Abstract

Patient-reported outcomes are information about health status and health-related quality of life collected directly from patients. The data in this publication contain the first assessment of patient-reported outcomes (PROs) from real-life measurements in the breast cancer center at Charité – Universitätsmedizin Berlin between November 2016 and March 2021.

At baseline (before the start of treatment), 1727 ambulatory patients with early breast cancer, ductal carcinoma *in situ* (DCIS), fibroadenoma, and other breast diseases were registered in the digital PRO-system as part of clinical routine. Patients’ sociodemographic data, medical history, clinical variables, and raw scores of the EORTC QLQ-C30 and EORTC QLQ-BR23 are provided in this publication.

This dataset can be used as a reference for PROs in a clinical care setting or in clinical studies with breast diseases and contribute to the discussion about the interpretation of score values.

Furthermore, the association between patients’ sociodemographic data, clinical variables, and PRO data at baseline can be analysed further.

## Specifications Table

**Table:** 

Subject	Health and medical sciences, Oncology
Specific subject area	Patient-reported outcomes (PROs)Breast cancerDuctal carcinoma *in situ* (DCIS)FibroadenomaOther breast diseasesHealth-related quality of lifeValue based healthcare
Type of data	Table
How the data were acquired	The data were acquired prospectively from patients with breast diseases via digital surveys at baseline. The surveys contained questionnaires on medical history and sociodemographics (anamnesis at initial admission) and Patient-reported outcomes (EORTC QLQ-C30 and EORTC QLQ-BR23). Clinical data were documented afterwards in the PRO system by doctoral students or student assistants based on medical files. All data are stored together in a web-based PRO-system (Heartbeat Medical, Germany).
Data format	RawFiltered
Description of data collection	The data collection process began in November 2016 and included all new patients in the outpatient breast clinic at Charité – Universitätsmedizin Berlin who gave consent to participate in the PRO program. Patients were asked to answer PROs (EORTC QLQ-C30 and EORTC QLQ-BR23) and provide anamnesis data (medical history and sociodemographic) on a tablet computer in the waiting room prior to their appointment. Clinical data were completed later by doctoral students or student assistants based on hospital files.The inclusion criteria for this dataset were as follows: - Patients with diagnosis of breast cancer, DCIS, fibroadenoma, or other breast diseases - Age 18 or over - German speakers The exclusion criteria were as follows: - Non-eligible diagnosis - Diagnosis date after March 31, 2021 - Missing diagnosis or diagnosis date - Diagnosis changed into non-eligible diagnosis type (e.g., diagnosis date > 7 days after initial survey) - First survey after treatment initiation - Second opinion
Data source location	• Institution: Department of Gynecology with Breast Center, Charité – Universitätsmedizin Berlin • City: Berlin • Country: Germany
Data accessibility	Repository name: Mendeley DataData identification number: https://doi.org/10.17632/wrhr5862cb.4Direct URL to data: https://data.mendeley.com/datasets/wrhr5862cb/4
Related research article	Karsten, M. M., Roehle, R., Albers, S., Pross, T., Hage, A. M., Weiler, K., Fischer, F., Rose, M., Kühn, F., & Blohmer, J. U. (2022). Real-world reference scores for EORTC QLQ-C30 and EORTC QLQ-BR23 in early breast cancer patients. European journal of cancer (Oxford, England: 1990), 163, 128–139. Advance online publication. https://doi.org/10.1016/j.ejca.2021.12.020

## Value of the Data

• This data provide reference scores for EORTC QLQ-C30 and the EORTC QLQ-BR23 in patients with early breast cancer and other breast diseases from real-life clinical care.
• It can be reused for further research regarding the relationship between patient demographics, clinical variables and PRO data. Further, one can potentially identify patterns of patients with a need for closer monitoring and symptom control before the start of treatment.
• Since standardized thresholds for critical PRO scores in breast diseases and minimally important clinical differences (MCID) are not well defined, this data can contribute to the discussion.
• There needs to be a discussion since defining normal values for sick patients remains challenging. Therefore, comparisons of PRO scores of patients with breast cancer should not stay limited to the normal population. Data of patients with benign breast diseases might also be valuable reference data for the interpretation of scores in patient care.
• This project emphasizes the importance of assessing PROs in routine clinical settings and demonstrates their feasibility.
• Collecting PROs serves as a foundation for value-based healthcare.

## 1 Data Description

We present supporting data to the research article “Real-world reference scores for EORTC QLQ-C30 and EORTC QLQ-BR23 in early breast cancer patients” by Karsten et al [1]. This section describes how the data are provided in the Mendeley Data repository, including the raw data, data dictionary, and questionnaire.

### 1.1 Raw Data

Raw data are provided in an Excel table and contains the following datasets:

#### 1.1.1 Anamnestic Dataset: Sociodemographic Data and Medical History

The anamnestic dataset consists of sociodemographic data and the medical history of the study's population. Sociodemographic data include the age in years at the time of registration, marital status, education, the frequency of alcohol consumption and the smoking status. The medical history data describe the current body mass index and various comorbidities, as well as a detailed gynaecological status. This includes bust measurement, cup size, age at time of menarche, number of pregnancies and births, use of contraceptives, if menopause has already occurred, and family history of breast or ovarian cancer. In addition, it indicated whether the current breast disease is the first breast disease that has occurred in the patient, and whether there has been previous breast surgery.

#### 1.1.2 Clinical dataset

The clinical dataset contains information about the affected breast side and the corresponding breast disease: breast cancer, ductal carcinoma in situ (DCIS), fibroadenoma, or other breast disease. If applicable, histological type, tumor grading, and the receptor status for estrogen, progesterone, and HER2/neu are provided.

#### 1.1.3 Health-Related Quality of Life Dataset

The health-related Quality of Life dataset consists of the European Organization for Research and Treatment of Cancer (EORTC) QLQ-C30 [2] and the breast cancer-specific supplement EORTC QLQ-BR23 [3] in raw data form in conformity with the EORTC manual [4]. The EORTC-QLQ-C30 lists the variables global health status/QoL, physical functioning, role functioning, emotional functioning, cognitive functioning, social functioning, financial difficulties, as well as specific symptom variables, namely fatigue, nausea and vomiting, pain, dyspnea, insomnia, appetite loss, constipation, and diarrhea. The EORTC-QLQ-BR23 includes variables specific for breast cancer, including arm symptoms, body image, breast symptoms, future perspective, sexual enjoyment, sexual functioning, system therapy side effects, and upset by hair loss.

### 1.2 Data Dictionary

A data dictionary is available in the form of an Excel table for all anamnestic, clinical, and health-related quality of life datasets. It contains the name and description of each variable, including data type (e.g., integer, nominal, decimal), data coding, data label, and remark.

### 1.3 Study Questionnaire

Only the sociodemographic questionnaire is provided in this study, and the study questionnaire is available in pdf format. It includes information on each question as well as the coding for possible responses.

**Fig. 1:**
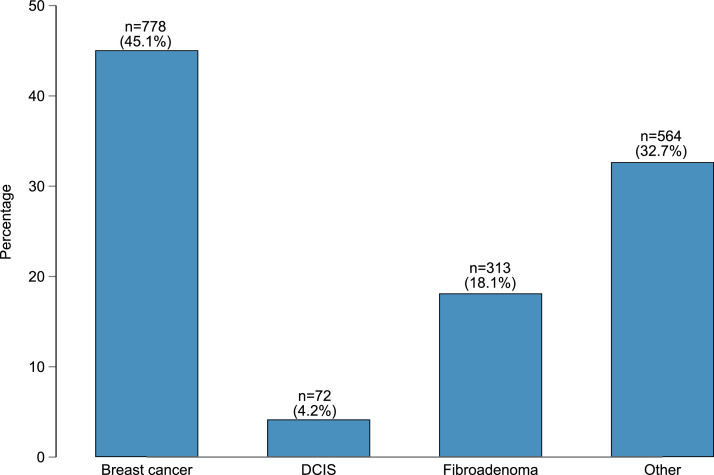
A bar chart of the distribution of breast cancer, DCIS, fibroadenoma, and other breast diseases in the study population (n = 1727 patients), including total numbers (n) and percentages (%).

Additional, the distribution of patients by breast diseases is presented by bar chart, including patients with early breast cancer (n = 778), DCIS (*n* = 72), fibroadenoma (*n* = 313) as well as other breast diseases (mastopathy, cysts, lipoma, phyllodes tumor, papilloma) (*n* = 564) (Fig. 1).

**Fig. 2:**
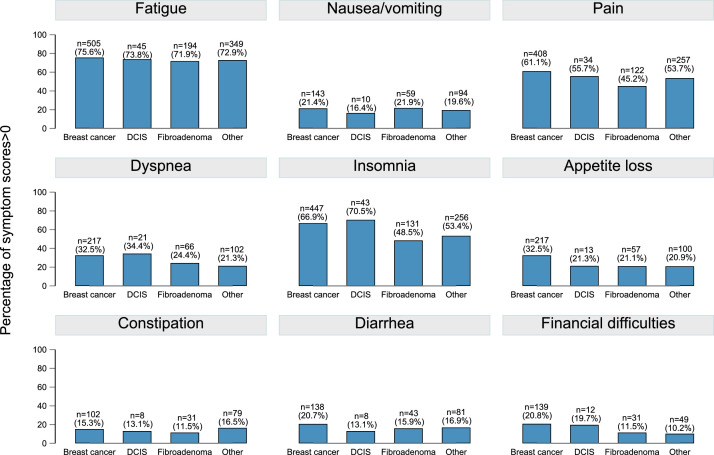
The numbers (n) and percentages (%) of patients who reported symptom scores of the EORTC QLQ C-30 greater than 0 among patients with breast cancer (total *n* = 668), DCIS (total *n* = 61), fibroadenoma (total *n* = 270), and other breast diseases (total *n* = 479) at baseline. Fatigue was observed by more than 70% of breast cancer patients across all diagnosis groups. In comparison to patients with fibroadenoma and other breast illnesses, patients with breast cancer and DCIS reported higher pain and insomnia. Only few patients reported symptoms of nausea and vomiting, dyspnea, appetite loss, constipation, diarrhea, and financial difficulties in all groups of patients.

At baseline, 1478 of the patients who were included in this study answered the PRO assessment (breast cancer = 668, DCIS = 61, fibroadenoma = 270, and other breast diseases = 479. Fig. 2 presents the symptom scores>0 of the EORTC QLQ C-30 separated by breast diseases and symptoms.

## 2 Experimental Design, Materials and Methods

Ambulatory patients visiting the breast cancer center at Charité – Universitätsmedizin Berlin between November 2016 and March 2021 were asked to participate in the PRO program as part of clinical routine [5]. The assessment was performed electronically on tablet computers in the waiting area of the outpatient clinic. All surveys were provided in German via a web-based PRO-system (Heartbeat Medical).

The questionnaires included PROs and anamnesis (medical history and sociodemographic data). PROs were collected using EORTC QLQ-C30, a 30-item instrument for measuring health-related quality of life (HRQoL) in oncologic patients and EORTC QLQ-BR23, a 23-item breast cancer specific supplement. Both instruments use four-point Likert scales, ranging from 1 (not at all) to 4 (very much) and a seven-point scale for global health status and QoL (GL).

The anamnestic questionnaire is based on the ICHOM breast cancer standard set [6] but has been modified to German standards by the study group. Additionally, clinical data were documented afterwards in the PRO system by doctoral students or student assistants based on medical files.

A total of 3689 patients participated in the PRO assessment in the studied time period. Out of these, 1727 patients (48%) were eligible for this dataset describing scores at baseline of patients with breast diseases [1]. Most of them did not know their definitive diagnosis before the assessment of PROs [6] and all of them were before the start of their initial treatment. If patients were diagnosed with more than one breast disease, they were labelled according to the following algorithm: breast cancer/DCIS > fibroadenoma > other breast disease. Patients who were not treated at Charité and only visited the outpatient clinic for a second opinion were excluded.

The PRO program at Charité is ongoing and prospectively follows-up on patients after their baseline participation.

Descriptive statistics (number of patients and percentages) were presented. The symptom scores of the EORTC QLQ C-30 were grouped into two groups (score = 0 and score > 0). All figures were created using Stata IC15 (StataCorp, 2017, College Station, TX, USA).

## Ethics Statements

The routine web-based PRO assessment was approved by the ethics committees at Charité – Universitätsmedizin Berlin (Ethics protocol number: EA4/127/16). The participants were enrolled in the study after providing written informed consent.

## CRediT authorship contribution statement

**Pimrapat Gebert:** Data curation, Formal analysis, Writing – original draft, Visualization. **Adam David Dordevic:** Writing – original draft. **Robert Roehle:** Data curation, Writing – review & editing. **Anna Maria Hage:** Project administration, Data curation, Writing – original draft. **Maria Margarete Karsten:** Conceptualization, Methodology, Writing – review & editing, Project administration, Supervision, Funding acquisition.

## Declaration of Competing Interest

The authors declare that they have no known competing financial interests or personal relationships that could have appeared to influence the work reported in this paper.

## Data Availability

Data PROM baseline (Original data) (Mendeley data).
